# Does dutasteride reduce perioperative blood loss and postoperative complications after transurethral resection of the prostate?

**Published:** 2007

**Authors:** R. Shanmugasundaram, J. Chandra Singh, Nitin S. Kekre

**Affiliations:** Department of Urology, Christian Medical College, Vellore, India. E-mail: uro2@cmcvellore.ac.in

## SUMMARY

The primary objective of this multicentric prospective randomized trial[1] was to determine whether pretreatment with dutasteride, a dual 5α-reductase inhibitor (5ARI), reduces surgical blood loss in patients who undergo transurethral resection of the prostate (TURP) for benign prostatic hyperplasia (BPH) with prostates ≥ 30 ml volume. The secondary objectives were to assess bleeding after TURP and postoperative complications like clot retention, blood transfusions, urinary tract infection (UTI), incontinence and acute urinary retention (AUR) following TURP. Two hundred and thirteen men aged 52–85 years (mean 67) with prostate volume of ≥ 30 ml were recruited from 23 centers across six countries. Two hundred and two had TURP and 197 completed the study. They were randomized to three groups: Group I receiving placebo for four weeks preoperatively and two weeks postoperatively, Group II receiving placebo for two weeks followed by dutasteride 0.5 mg for two weeks preoperatively and two weeks of dutasteride 0.5 mg daily postoperatively and Group III with dutasteride 0.5 mg four weeks preoperatively and two weeks postoperatively. Prior prostate surgery, history or evidence of prostatic disease other than BPH, those who had 5ARI within 12 months, those on aspirin and NSAIDS and those with severe medical conditions like liver disease, unstable cardiovascular diseases and bleeding disorders were excluded. All the patient events four weeks before and four weeks after TURP were recorded using daily diary. Subsequently, weekly diary was maintained for 10 weeks. Blood loss was measured from irrigation fluid drained using HemoCue low hemoglobin system-photometer. Serum testosterone, dihydrotestosterone (DHT) were measured during recruitment, during TURP and four weeks after TURP. Microvessel density (MVD) and intraprostatic levels of hormones were measured from prostatic chips after TURP. The mean operative duration was 45 min and about 25 g of prostate was resected. Mean blood loss during TURP was 2.15 to 2.55 gm Hb/gm of resectate in the three groups. There were no statistically significant differences in the blood loss during surgery among the treatment groups. Hemoglobin loss after TURP was about a third of the total surgical blood loss. one to three per cent of the patients in each group received blood transfusions. Clot retention occurred in 6-11% of the patients, AUR in 11-17%, UTI in 20-31% and urinary incontinence in 14-15% of the patients over 14 weeks, but there was no statistically significant differences among groups. Treatment with dutasteride did not alter the MVD in the prostatic urethra or in regions of nodular hyperplasia. The drug reduced the serum concentration of DHT by 86-89% (*P*< 0.001), while increasing serum testosterone level by 13-15% before treatment. Dutasteride also reduced the intraprostatic concentration of DHT by 10 times compared with placebo. No adverse events that were considered to be drug-related were recorded.

## COMMENTS

5ARIs have been popularized during the perioperative period to reduce blood loss during TURP for larger glands (>30 ml). Though these drugs shrink the prostate by attenuating the blood vessels, the results of studies of 5ARIs on blood loss during TURP have been inconsistent and reasons have been stated. Pretreatment with finasteride for duration of two weeks,[2] eight to 10 weeks[3] had been shown to reduce bleeding during TURP for larger glands. However, Sandfeldt *et al.*[4] observed that there was no difference in blood loss intraoperatively and perioperatively even after three months of pretreatment with finasteride on 30 to 90 cm^3^ prostates. The multicentric randomized placebo-controlled trial by Boccon-Gibod *et al.*[5] to study the effect of pretreatment with dutasteride 0.5mg for four weeks prior to TURP showed no significant difference between the treatment groups. The majority of the studies showing that 5ARIs are beneficial in reducing blood loss during TURP are single center trials and having less number of patients than the present study.

This multicentric study concludes that pretreatment with dutasteride 0.5 mg for two weeks, four weeks and continuing it postoperatively for two weeks did not reduce blood loss and related complications significantly when compared to placebo in spite of reducing intraprostatic concentrations of DHT. There is variation in the baseline mean prostate volume among the three groups, the largest mean being in the dutasteride group. The complication rates seem to be higher than the contemporary series[6] but the authors feel it is probably due to the prospective documentation of these events. Standardizing the operative procedure and ensuring that each center has similar distribution of the three groups will minimize the impact due to variation in techniques. Further studies incorporating these issues are likely to give a more emphatic verdict on this issue.
